# Role of omega-3 polyunsaturated fatty acids, citrus pectin, and milk-derived exosomes on intestinal barrier integrity and immunity in animals

**DOI:** 10.1186/s40104-022-00690-7

**Published:** 2022-04-11

**Authors:** Tamil Selvi Sundaram, Carlotta Giromini, Raffaella Rebucci, Juraj Pistl, Mangesh Bhide, Antonella Baldi

**Affiliations:** 1 Department of Veterinary Science for Health, Animal Production and Food Safety, University of Milan, Via Trentacoste 2, 20134 Milan, Italy; 2 University of Veterinary Medicine and Pharmacy in Košice, Komenského 68/73, 04181 Košice, Slovakia

**Keywords:** Citrus pectin, Immunomodulatory nutrients, Inflammation, Intestinal epithelial barrier, Milk-derived exosomes, Omega-3 polyunsaturated fatty acids, Oxidative stress

## Abstract

The gastrointestinal tract of livestock and poultry is prone to challenge by feedborne antigens, pathogens, and other stress factors in the farm environment. Excessive physiological inflammation and oxidative stress that arises firstly disrupts the intestinal epithelial barrier followed by other components of the gastrointestinal tract. In the present review, the interrelationship between intestinal barrier inflammation and oxidative stress that contributes to the pathogenesis of inflammatory bowel disease was described. Further, the role of naturally existing immunomodulatory nutrients such as the omega-3 polyunsaturated fatty acids, citrus pectin, and milk-derived exosomes in preventing intestinal barrier inflammation was discussed. Based on the existing evidence, the possible molecular mechanism of these bioactive nutrients in the intestinal barrier was outlined for application in animal diets.

## Introduction

Apart from nutrient absorption, the intestinal epithelial layer (IEL) acts as the first line of host defense against various environmental stress factors. Excessive physiological inflammation and oxidative stress primarily disrupts the inner lining of the gastrointestinal tract such as the IEL. Impairment of IEL can further contribute to the progression of inflammatory bowel diseases (IBD), metabolic disorders, and even mortality in animals [1–3]. Traditionally antibiotics are administered in animal diets to enhance the growth performance and prevent undesired inflammatory responses. Although antibiotics are beneficial to a certain extent, the development of antibiotic-resistance in bacterial populations is a major drawback [4, 5]. Hence, there is a necessity for alternative, environmental-friendly immunomodulatory compounds to nurture animal health with limited side effects. Of note, certain bioactive compounds derived from plants, animals, and microbial sources exhibit therapeutic benefits beyond basic nutrition. The addition of these compounds to a certain level in an animal diet is reported to boost the gut immunity, confer stress resistance, and improve the overall health status [3, 6, 7]. The examples of such potential bioactive compounds include the traditional and emerging omega-3 polyunsaturated fatty acids (ω-3 PUFAs), citrus pectin (CPn), and the milk-derived exosomes (MDEs). In the previous in vitro and in vivo trials, these natural nutrients exhibited strong immunomodulatory, antioxidative, and antimicrobial properties in controlling chronic gut inflammation. This review describes the impact of inflammation and oxidative stress in the intestinal barrier that contributes to IBD. Further, the role of immunomodulatory nutrients as the ω-3 PUFAs, CPn and MDEs in controlling gut inflammation at the level of intestinal barrier is discussed for utilization in animal nutrition.

## Intestinal epithelium – a dynamic barrier

**Fig. 1:**
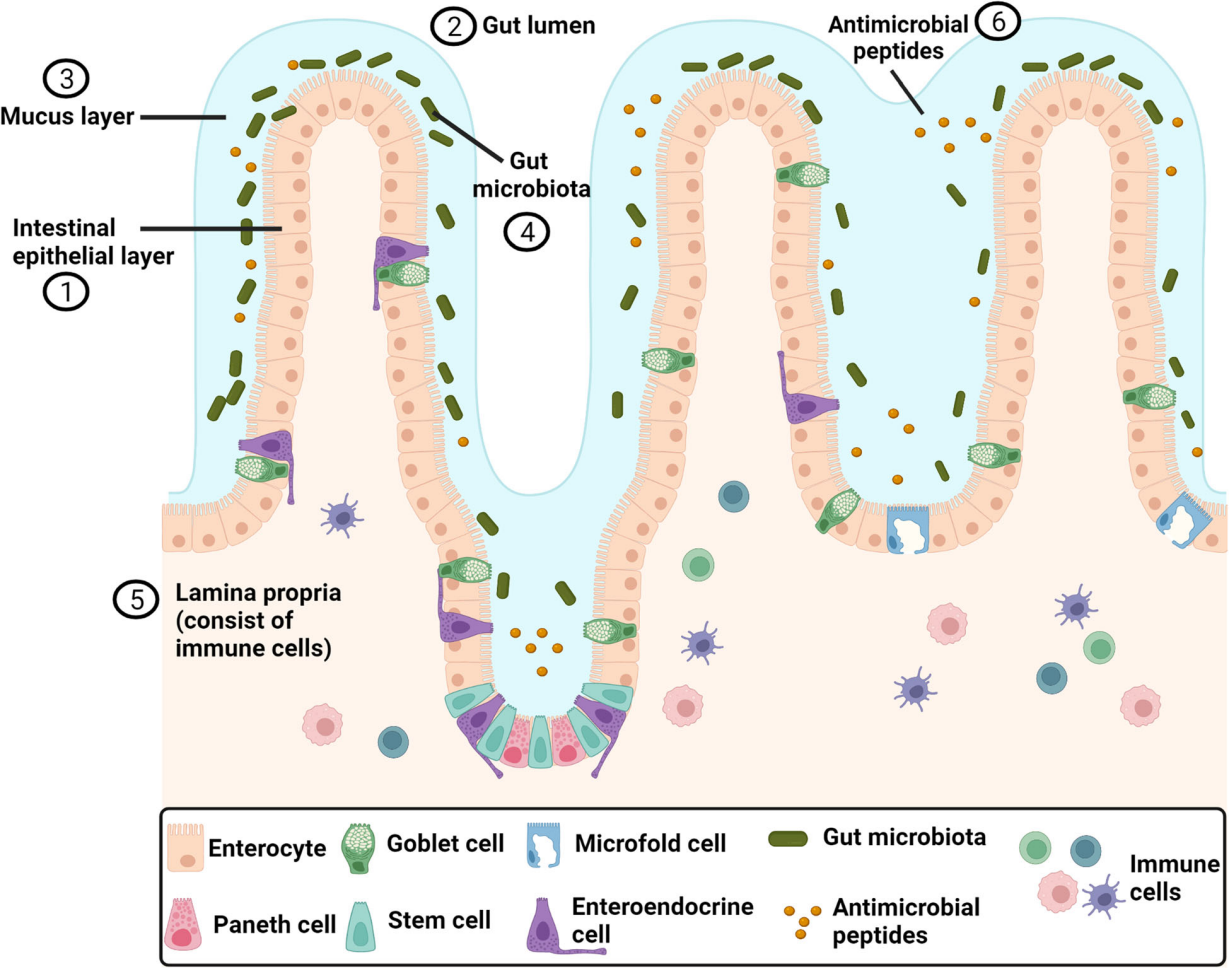
Structure of intestinal epithelium. The intestinal epithelial layer (IEL) ① is the first lining of gastrointestinal tract. It is formed by a single layer of specialized intestinal epithelial cells (enterocytes, goblet cells, paneth cells, microfold cells, stem cells and enteroendocrine cells) that physically separates the gut lumen ② from the circulatory system. The IEL is lined by a mucous layer ③, where the gut microbiota ④ is embedded. The IEL orchestrates gut homeostasis by establishing communication between the gut microbiota and the underlying immune cells in lamina propria ⑤. The intestinal epithelial cells secrete several antimicrobial peptides ⑥ for host-defense. For references, see text. Figure created using BioRender.com

Gastrointestinal tract is secured by a series of protective layers collectively described as the intestinal mucosa. It mainly encompasses the mucus layer, gut microbiota, IEL, immune cells dispersed in lamina propria and lamina. The IEL physically separates the circulatory system from the external milieu [Fig. 1]. Besides, it is the key component that orchestrates gut homeostasis by establishing communication between the microbiota and the underlying immune cells [8, 9]. Further, the IEL is formed by a monolayer of intestinal epithelial cells (IECs) interconnected by different protein complexes such as tight junction proteins, junction adherent proteins (JAM) and, desmosomes. The tight junction proteins such as occludens (OCLN) and claudins (CLDN) are the important structural units that strictly governs the permeability of molecules across the barrier [10]. Besides, the IEL comprises several specialized absorptive and secretory cell types for digestion, nutrition uptake, and host defense. Particularly, the enterocytes are the most abundant (~ 80%) and fast regenerating absorptive cell type [11, 12]. Goblet cells secrete a gel-like glycoproteins called mucin (MUC) that forms a mucous layer above the IEL. Further, the mucus layer is embedded with trillions of commensal bacteria called the gut microbiota. Paneth cells exhibit the longest life span and produce antimicrobial proteins such as defensins, lysozymes, and phospholipases [10, 13]. The microfold cells regulate the adaptive immune responses by presenting the luminal pathogens and antigens to the immune cells of lamina propria [10]. Enteroendocrine cells primarily secrete hormones and other antimicrobial proteins for different physiological functions. The multipotent intestinal stem cells at the crypt base continuously regenerate to form other specialized cell types [11]. Altogether, the IEL forms a dynamic barrier that selectively permits the nutrients while block the detrimental factors as pathogens and toxins from entering into the circulatory system.

## Implications of inflammation and oxidative stress in intestinal barrier

Gastrointestinal tract is prone to inflammatory and oxidative damages owing to continuous contact with environmental stress factors. IECs exhibit specialized pattern-recognizing receptors (PRRs) such as the toll-like receptors (TLRs) and nucleotide oligomerization domain (NOD)-like receptors for identification of pathogen-associated molecular patterns (PAMPs) or damage-associated molecular patterns (DAMPs) [14]. Subsequently, the IECs secrete various antimicrobial proteins and pro-inflammatory signaling mediators such as cytokines, chemokines, and reactive oxygen species (ROS). These mediators are responsible for the differentiation, maturation, and activation of other cells in the immune system [15, 16]. Generally, oxidative stress can occur when an imbalance exists between the generation of ROS and the antioxidants available to scavenge [17]. ROS can directly damage the proteins, DNA, and lipids [17] or indirectly control certain transcription factors such as the nuclear factor-κB (NF-κB) [18]. NF-κB was described as the chief stimulator of infection and inflammation that activates more than 200 genes of pro-angiogenic factors, pro-inflammation, apoptosis, and inducible enzymes [19]. These observations explain the relationship between inflammation and oxidative stress, that can trigger one another interchangeably [18]. To a certain degree, physiological stress is necessary, however, in excess is detrimental. Occasionally, uncontrolled inflammation or oxidative stress can arise from defects in receptors, signal transduction, or during the de novo biosynthesis of pro-resolution mediators [20]. Particularly, high local concentrations of cytokines such as the tumor necrosis factor (TNF)-α, interleukin (IL)-1β, and IL-6 were reported to compromise the IEL by downregulating the tight junctions and JAMs [21]. Subsequently, the leaky IEL stimulates leukocyte transmigration, disrupts barrier architecture and IEC maturation [14]. Observations from human and animal studies have reported barrier defects to precede chronic inflammatory diseases [1]. Therefore, the integrity of IEL is of paramount importance in the maintenance of gut but also the overall health.

## Immunomodulatory nutrients for intestinal health

The pursuance of reducing the antibiotics and non-steroidal anti-inflammatory drugs (NSAIDs) has raised the interest in utilizing naturally existing bioactive compounds. These bioactive compounds are constituents of functional food, similar to conventional foods but provide additional therapeutic benefits. Thus, functional foods are described as the food prepared using ‘scientific intelligence’ vital for optimal health [6]. The European Food Safety Authority (EFSA) has categorized these natural bioactive or immunomodulatory compounds into, ‘plant extracts’, ‘prebiotics’, ‘probiotics’, ‘animal-by products’, and ‘other substances’ [22]. Plants have been the major source of energy since time immemorial in humans and animals. Certain compounds from whole or part of the plant exhibit immunomodulatory properties and are commonly referred to as ‘phytobiotics’ or ‘botanicals’ [23]. A classic example of a plant- or marine-derived immunomodulatory compound is the essential fatty acid such as the ω-3 PUFAs. The common sources of ω-3 PUFAs are marine phytoplankton, linseed oil, and fish oil [24, 25]. Since several decades, ω-3 PUFAs are routinely incorporated into human and animal diets in moderation for normal physiological functions. However, supplementation at a certain level result in achieving therapeutic effects. The ω-3 PUFA-enriched diet in livestock and poultry was shown to modulate the immune system, improve intestinal morphology, fertility, stress resistance, and performance [26–29].

The pectins from citrus fruit peels are increasingly recognized for its multiple health-beneficial properties. Although the application of CPn as a feed additive is emerging, preliminary studies have reported to exhibit potential anti-inflammatory, antimicrobial and prebiotic properties [30–33]. Besides, the food processing industries utilize CPn as gelling agent and stabilizers for ages and is regarded safe for consumption [34]. Moreover, GCS-100 and PectaSol-C are the two commercially available CPn-based drugs used for treating fibrosis in humans [35]. In the European Union, Italy is one of the leading producers of citrus fruits and after juicing, the peels are discarded as waste. These environmental wastes can otherwise be utilized as value-added, sustainable dietary supplements for animals. Another prominent, animal-based nutritive compound is the milk-derived bioactive peptides and proteins [36]. Recently, the exosomes present in milk was identified to carry cargoes of nucleic acids and peptides that improves the immune system, promotes intestinal barrier development, and prevent inflammatory diseases [37–40]. Currently, the milk-derived exosomes (MDEs) are gaining attention for targeted inflammatory diseases through diet [40]. Unlike antibiotics, these natural bioactive compounds are effective, environmentally friendly, and safe for improvising the immune system of animals, while eliminating stress and discomfort. The immunomodulatory properties of ω-3 PUFAs, CPn, and MDEs at the intestinal-level, based on previous evidence are described in the following sections.

### Omega-3 polyunsaturated fatty acids

#### Structure and molecular mechanism

**Fig. 2:**
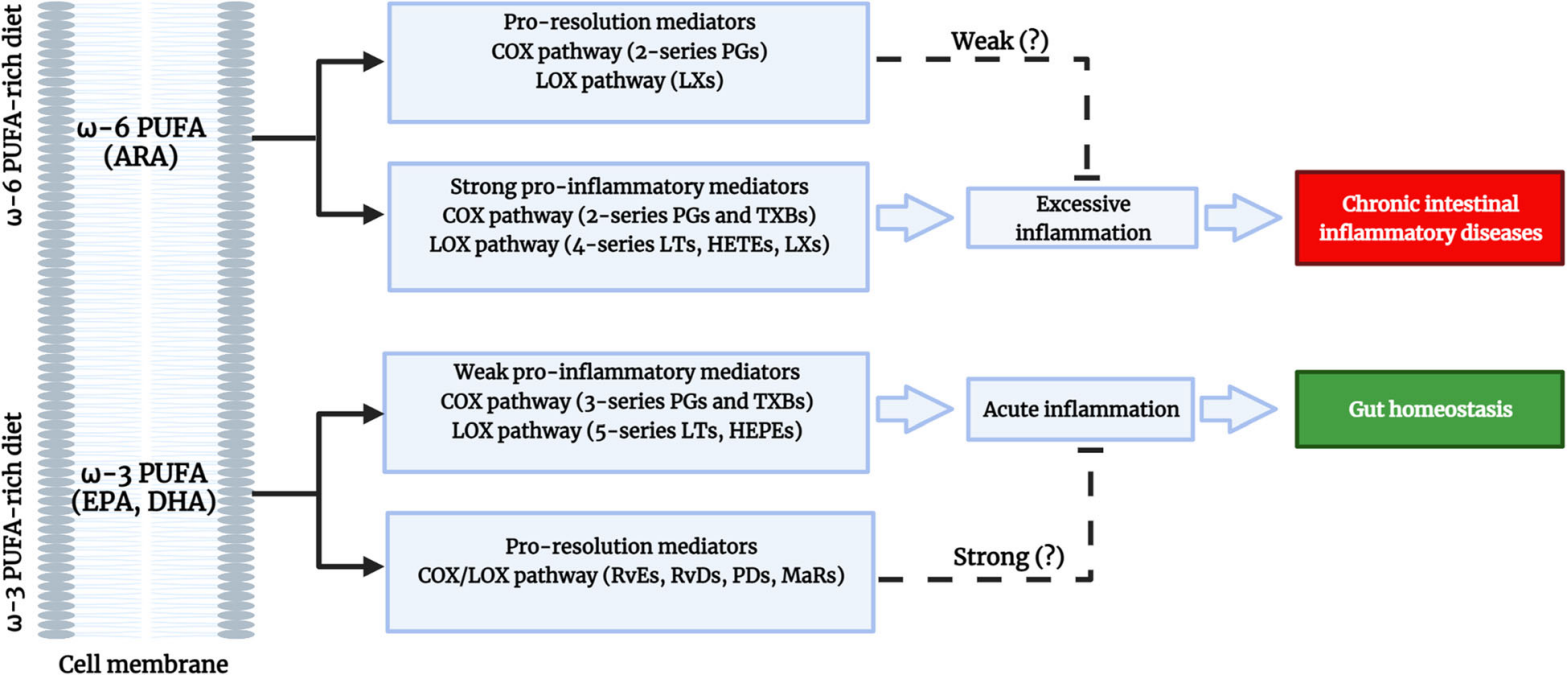
Eicosanoid families of omega-3 and omega-6 polyunsaturated fatty acids involved in intestinal inflammation and resolution. Eicosanoids are predominantly generated from the ω-6 arachidonic acid (ARA) in phospholipid cell membranes by the enzymatic actions of cyclooxygenase (COX) and lipoxygenase (LOX). The COX pathway generates 2-series prostaglandins (PGs) and thromboxanes (TXBs), while the LOX generates lipoxins (LXs), hydroxyeicosatetraenoic acids (HETEs) and, 4-series leukotrienes (LTs) that occasionally stimulates excessive pro-inflammatory response leading to chronic intestinal inflammatory diseases. On the other hand, the ω-3 eicosapentaenoic acid (EPA) stimulates acute inflammation by generating 3-series PGs and TXBs via the COX pathway, while hydroxyeicosapentaenoic acids (HEPEs) and 5-series LTs via the LOX pathway. Recently, certain ARA-derived PGs and LXs were identified to exert both pro-inflammatory and pro-resolution characteristics. Similarly, the newly identified downstream molecules of EPA such as the E-series resolvins (RvEs) and docosahexaenoic acid (DHA) such as the D-series resolvins (RvDs), maresins (MaRs), and protectins (PDs) are involved in pro-resolution. Both ω-3 and ω-6 polyunsaturated fatty acids (PUFA) supports pro-resolution, but overwhelming data reported ω-3 PUFA as the strongest anti-inflammatory agent. This could be due to the difference in magnitude of action among the eicosanoids involved in the resolution phase of inflammation. For references, see text. Figure created using BioRender.com

Although ω-3 PUFAs are widely known feed additive, its mechanism in intestinal barrier is poorly established, which limits the assessment of its efficacy. Based on previous studies, its possible modes of action are described presently. Generally, fatty acids are carbon chain structures of varying length, synthesized in the cytoplasm from Acetyl-CoA [41]. The two major fatty acid families as the omega-3 (ω-3) alpha-linolenic acid (ALA; C18:3n-3) and the omega-6 (ω-6) linoleic acid (LA; C18:2n-6) that cannot be synthesized by animals, but obtained from the diet are called essential fatty acids [24, 42]. Basically, PUFAs are involved in cell signaling, immunomodulation, and formation of structural components in the phospholipid cell membranes [43, 44]. An infection or injury triggers hydrolysis of PUFA from the cell membranes to release certain lipid-based signaling molecules called eicosanoids [45]. The family of eicosanoids orchestrate the initiation, progression, and resolution phases of the inflammatory responses. Owing to the higher dietary ratio of ω-6/ω-3 PUFAs in animals, the eicosanoids are predominantly generated from ω-6 arachidonic acid (ARA; C20:4n-6) by the enzymatic actions of cyclooxygenase (COX) and lipoxygenase (LOX) [45–47]. The COX (COX-1/-2/-3) pathway generates 2-series prostaglandins (PGs) and thromboxanes (TXBs), while the LOX (5-/12-/15-LOX) generates hydroxyeicosatetraenoic acids (HETEs), lipoxins (LXs), 4-series leukotrienes (LTs), and other oxidative derivatives [47, 48]. Similarly, the downstream derivatives of ω-3 ALA, specifically the eicosapentaenoic acid (EPA; C20:5n-3) generates 3-series PGs and TXBs via the COX-2 pathway, while hydroxyeicosapentaenoic acids (HEPEs) and 5-series LTs via the LOX (5-/12-/15-LOX) pathway [Fig. 2]. The ARA-derived pro-inflammatory eicosanoids are widely reported to alter the gut microbial composition, disrupts the intestinal barrier and play a central role in the pathogenesis of IBD [24, 45, 49]. Therefore, NSAIDs primarily target the inhibition of ARA and its derivatives mainly in the COX pathway. Conversely, EPA and docosahexaenoic acid (DHA; C22:6n-3) can replace ARA in the cell membranes and produce 100-fold weak pro-inflammatory eicosanoids for enhanced resolution of inflammation [50–54]. Furthermore, the newly identified downstream molecules of EPA such as the E-series resolvins (Rv)Es and DHA such as the D-series RvDs, maresins (MaRs), and protectins (PDs) act as antagonists of inflammation [20, 52, 53, 55] [Fig. 2]. These molecules not only terminate inflammation but scours inflammatory debris and stimulates antimicrobial defense for gut homeostasis [20]. Moreover, recently ARA-derived PGs (PGD2, PGE2) and LX(A4) were identified to exert both pro-inflammatory and pro-resolution characteristics in a process called, ‘class-switching’ [20, 52, 55]. These key findings eventually enumerate the fact that both ω-3 and ω-6 PUFAs generate anti-inflammatory eicosanoids and supports pro-resolution. However, overwhelming data denotes ω-3 PUFA as the strongest anti-inflammatory agent compared to ω-6 PUFA [21, 25, 43, 50]. This could possibly due to the difference in magnitude of action among the eicosanoids involved in resolution phase [Fig. 2]. Therefore, further studies are necessary to delineate the potency of pro-resolution eicosanoids to address the rationale behind ω-3 PUFA’s heightened anti-inflammatory action.

#### Impact on intestinal barrier under normal and stress conditions

##### In vitro studies

The impact of ω-3 PUFAs on the intestinal barrier under normal and inflammatory conditions were previously evaluated using different IEC models (Table 1). Particularly, the enterocyte models were widely utilized as they are primarily involved in nutrition absorption, host-defence and are the most abundant cell type in IEL. In these experimental models, the pathophysiological events of barrier inflammation and oxidative stress was stimulated using different biological and chemical stressors. Some commonly applied stressors are pathogens, endotoxins, chemicals such as acetic acid, hydrogen peroxide (H_2_O_2_), dextran sulfate sodium (DSS), and 2,4,6-trinitrobenzene sulfonic acid (TNBS) [66, 86]. Moreover, these models were widely reported to mimic the cardinal signs of IBD [86]. Accordingly, ω-3 PUFA (ALA or EPA) treatment modulated the integrity and permeability of human Caco-2 cell monolayer in a dose-dependent manner [56, 57]. The epithelial integrity was determined by measuring the transepithelial electrical resistance (TEER) across the monolayer of IECs. Whereas, the barrier permeability was assessed by measuring the paracellular flux of small molecules such as horseradish peroxidase (HRP), fluorescein-5-(6)-sulfonic acid (FS), or fluorescein isothiocyanate-labelled dextran 4 kDa (FD4) between the tight junction gaps in the cell monolayer [56–58]. Further, pre-incubation of human T84 cells with ω-3 PUFA (ALA, EPA or DHA) secured the monolayer integrity and permeability from the damage against IL-4 pro-inflammatory cytokine as indicated by increased TEER values and decreased FD4 permeability compared to the control [58]. Similarly, the negative impact of heat-injury in the human Caco-2 cell monolayer was reduced by ω-3 PUFA (EPA or DHA) pre-treatment. Specifically, EPA highly enhanced the barrier integrity (TEER) and expressions of different tight junction proteins (Zonula occludens [*ZO*]-1, *OCLN*, CLDN-2), while decreased the barrier permeability to HRP flux compared to DHA [59].

**Table 1:** Effects of omega-3 polyunsaturated fatty acids in the intestinal cell and animal models

Model of study	Stress by	ω-3 PUFA(s) assessed	Immune response and morphological changes	Reference
**In vitro**				
Human Caco-2 cells	Non-stimulated	EPA	(↓HRP flux, =TEER) at 100 μmol/L PUFA for 24 h; ↑LDH release > 100 μmol/L PUFA for 48 h	[56]
Human Caco-2 cells	Non-stimulated	ALA or EPA	(↑FS flux, ↓TEER) until 200 μmol/L PUFA; ↑LDH release for 200 μmol/L PUFA	[57]
Human T84 cells	IL-4	ALA, EPA or DHA	(↑TEER, ↓FD4 flux) for 100 μmol/L PUFA	[58]
Human Caco-2 cells	Heat injury	EPA or DHA	↑(TEER, *ZO-1*, *OCLN*, CLDN-2); ↓HRP flux	[59]
Rat IEC-6 cells	Mechanical wound	ALA, EPA, DHA or Docosapentaenoic acid	↑(TGF-β1, cell proliferation and migration in wound healing); ↓PGE2	[60, 61]
Human Caco-2 cells	IL-1β	ALA, EPA or DHA	↓(IL-6, IL-8, iNOS); ↑PPARγ; =IκB	[62]
Human Caco-2 cells	IL-1β	ALA or fish oil	↓(*COX-2*, *IL-8*, *iNOS*)	[63]
Human Caco-2 cells, human NCM460 cells, fetal H4 cells or neonate NEC-IEC cells	IL-1β	EPA or DHA	↓(*NF-κβ1*, *IL-8*, *IL-6*, *IL-1R1*)	[64]
Porcine IPEC-1 cells	DON	EPA or DHA	↑(Cell proliferation, viability, LDH release) until 25 μg/mL PUFA; ↑(TEER, CLDN-1, ZO-1) at 12.5 μg/mL PUFA; ↓(FD4 flux, caspase-3/-8, necrotic cells, ROS TNFR1, RIPK-1/-3, MLKL, PGAM5, DRP1, HMGB1) at 12.5 μg/mL PUFA	[65]
Porcine IPEC-J2 cells	LPS, DSS or H_2_O_2_	EPA or DHA	↑(Cell proliferation, viability); ↓(LDH release, caspase-3/-7); =NO_2_^−^	[66]
Pig ileum explants	LPS	Fish oil	↓LPS permeability; =TEER	[67]
**In vivo**				
Rat	Acetic acid-colitis	ω-3 PUFA-rich lipid emulsion	↓(PGE2, LTB4, TXB2, macrophage infiltration, mucosal and tissue damage); =LTC4	[68]
Rat	TNBS-colitis	ALA	↓(ICAM-1, VCAM-1, VEGFR-2); =HO-1	[69]
Rat	TNBS-colitis	Fish oil	↓(COX-2, PGE2, LTB4, NF-κB); =(*TNF-α, IL-1β*, PPARγ, mucosal damage, inflammatory cell infiltration)	[70]
Rat	DSS-colitis	Fish oil	↓(Disease activity, colon weight/length ratio, mucosal ulceration, crypt dilation, goblet cell depletion, inflammatory cell infiltration, tissue damage, MPO, iNOS, AP, COX-2, LTB4, TNF-α); ↑glutathione; =IL-1β	[71]
Rat	DSS-colitis	MaR1	↓(Disease activity, colon shortening, mucosal damage, inflammatory cell infiltration, PGE2, MPO, ROS, TLR4, p-NF-κB-p65, TNF-α, IL-6, IL-1β); ↑(ZO-1, OCLN, NRF2, HO-1)	[72]
Neonate rats	Hypoxia and formula feed-induced NEC	DHA	↓(*TLR2/4*, *PAFR*, tissue necrosis, incidence of disease); =phospholipase A2-II in small and/or large intestine	[73]
Maternal rats	Formula feed-induced NEC	EPA or DHA	↑(PGE2 receptor *EP3*, PGD2 receptor *DP2*, *PPARγ*) and ↓(*IκBα/β*, mucosal damage, inflammatory cell infiltration, incidence of disease) in premature rat pups	[74]
Mice	Acute or chronic DSS-colitis	MaR1	↓(Disease activity, MPO, NF-κB, IL-1β, IL-6, TNF-α, INFγ, *ICAM-1*, crypt damage, inflammatory cell infiltration, colon wall thickness, colon shortening, hyperaemia and tissue damage)	[75]
Peritonitis mice	TNBS-colitis	RvE1	↑Tissue repair; ↓(leukocyte infiltration, *COX-2*, *TNF-α, IL-12p40, iNOS,* MPO); =(*INF-γ*, *IL-4, IL-10, TGF-β*)	[76]
Mice	*Citrobacter rodentium*	Fish oil	↑IL-10; ↓(mucosal damage, inflammatory cell infiltration, apoptotic cells, Ki67^+^ enterocytes, *MIP-2,* keratinocyte cytokine, *MCP-1, IFN-γ, IL-6, IL-17A,* FD4 intestinal permeability); =(mucosal adherent pathogen count, *TNF-α, TGF-β*, *FoxP3*)	[77]
Mice	LPS	Fish oil	↓(*COX-2*, *TLR4, MyD88, NF-κB*, *IL-1β*, IL-6, TNF-α); =(*iNOS*, MCP-1, *IL6, TNF-α*) in small intestine	[78]
Mice	LPS	EPA or DHA	↑(E-cadherin, ZO-1, OCLN, *GPR120, FFAR-2*, *MUC2*, *IL-10*, tissue repair); ↓(NF-κB-p65, TAK1, *INFγ*, *TNF-α*, *IL-1β*, *IL-6*); =(TLR4, jejunum villus hight, crypt depth, V/C ratio)	[79]
Pigs	Non-stimulated	Fish oil	↓(PGE, PGI2, TXB2, crypt depth); =(colonic mucosal morphology)	[80]
Sows	Non-stimulated	Linseed oil	↓(δ-5/-6 desaturase, HRP flux until d 28, villus height at d 0, crypt depth at d 7) in piglet ileum; =ileum enterocyte maturation in piglets	[81]
Sows	Non-stimulated	Extruded linseed	↑(FD4/LPS permeability between d 0–28 in piglet jejunum explant); =(FD4/LPS permeability at d 52, IL-8, TNF-α in LPS-challenged piglet jejunum explants)	[82]
Weaned piglets	LPS	Fish oil	↑(Villus height, V/C ratio, OCLN, CLDN-1); ↓(diamine oxidase, TNF-α, PGE2, caspase-3, HSP70, NF-κB-p65, *TLR4, MyD88, IRAK1*, *TRAF6*, *NOD2*, *RIPK-2*); =(*NF-κB-p65*, *NOD1*, crypt depth) in jejunum and/or ileum	[83]
Weaned piglets	DSS-colitis	Fish oil	↑(Mitotic figures in enterocytes, *UPC3*, disease remission); =(mucosal damage, *PPARγ*, *PGC1-α*, *TNF-α*, *KGF*)	[84]
Suckling piglets	Ischemia-injured ileum	EPA	↓PGE2; ↑(TEER, H^3^-mannitol/C^14^-inulin flux, *COX-2*); =mucosal damage	[85]

The arrow indicates an increase (↑) or decrease (↓) in the level or activity of the different parameters analysed, “=” symbol designates unchanged parameters. *ALA* Alpha-linolenic acid, *AP* Alkaline phosphatase, *COX* Cyclooxygenase, *DHA* Docosahexaenoic acid; *DON*: Deoxynivalenol; *DRP1*: Dynamin-related protein 1, *DSS* Dextran sulphate sodium, *17,18-EEP* 17,18-Epoxyeicosatetraenoic acid, *EPA* Eicosapentaenoic acid, *FD4* Fluorescein isothiocyanate-labelled dextran 4 kDa, *FFAR-2* Free fatty acid receptor-2, *FS* Fluorescein sulfonic acid, *5-FU* 5-Fluorouracil, *GPR* G-protein coupled receptor, *HMGB1* High mobility group box-1 protein, *HO-1* Heme oxygenase-1, *H*_*2*_*O*_*2*_ Hydrogen peroxide, *HRP* Horseradish peroxidase, *HSP70* Heat shock protein-70, *IκB* Inhibitor of nuclear factor kappa B, *ICAM-1* Intercellular adhesion molecule-1, *IEC* Intestinal epithelial cells, *IL* Interleukin, *INFγ* Interferon γ, *iNOS* Inducible nitric oxide synthase, *IRAK1* Interleukin-1 receptor-associated kinase-1, *8-Iso PGF3α* 8-Iso prostaglandin F3α, *KGF* Keratinocyte growth factor, *LDH* Lactate dehydrogenase, *LPS* Lipopolysaccharides, *LT* Leukotriene, *MaR* Maresin, *MCP-1* Monocyte chemoattractant protein-1, *MIP-2* Macrophage inflammatory protein-2, *MLK*L Phosphorylated mixed lineage kinase-like protein, *MPO* Myeloperoxidase, *MUC* Mucin, *MyD88* Myeloid differentiation primary response 88, *NEC* Necrotizing enterocolitis, *NF-κB* Nuclear factor-κB, *NO*^*2−*^*/NO*^*3*−^ Nitrite/nitrate, *NOD* Nucleotide-binding oligomerization domain-containing protein, *NRF2* Nuclear factor erythroid 2-related factor 2, *OCLN* Occludin, PAFR Platelet-activating factor receptor, *PGAM5* Phosphoglycerate mutase family 5, *PG* Prostaglandin*, PGC1-α* PPARγ co-activator 1-α, *PPAR* Peroxisome proliferator-activated receptor, *PUFA* Polyunsaturated fatty acid, *RIPK* Receptor-interacting protein kinase, *ROS* Reactive oxygen species, *RvE* E-series resolvin, *TAK1* Transforming growth factor-β-activated kinase 1, *TEER* transepithelial electrical resistance, *TGF* Transforming growth factor, TNBS 2,4,6-trinitrobenzene sulfonic acid, *TNF* Tumour necrosis factor, *TNFR1* Tumour necrosis factor receptor-1, *TRAF6* Tumour Necrosis Factor Receptor-Associated Factor-6, *TXB* Thromboxane, *UPC3* PPARγ-responsive gene uncoupling protein-3, *V/C* Villus height/crypt depth, *VCAM-1* Vascular adhesion molecule-1, *VEGFR-2* Vascular endothelial growth factor-2, *ZO* Zonula occludens

In the mechanically injured rat IEC-6 cells, ω-3 PUFA (ALA, EPA, DHA, or docosapentaenoic acid) accelerated cell proliferation and wound healing by decreasing the level of pro-inflammatory PGE2 eicosanoid and instead enhanced the expression of transforming growth factor (TGF)-β1 [60, 61]. Further, ω-3 PUFA (ALA, EPA, DHA or fish oil) pre-treatment suppressed the expression of IL-1β-stimulated genes (*COX-2, IL-8,* inducible nitric oxide synthase [*iNOS*]) or proteins (IL-6/-8, iNOS) that involved in the pro-inflammatory response of Caco-2 cells and instead activated the protein expression of inhibitor of nuclear factor kappa B (IκB) and peroxisome proliferator-activated receptor(PPAR)γ [62, 63]. In another study, compared to EPA, DHA highly suppressed the expression of genes or proteins that involved in the pro-inflammatory response (*NF-κβ1, IL-1R1/-6/-8*) of human adult IECs (Caco-2 and NCM460 cells), fetal IECs (H4 cells), and neonate necrotizing enterocolitis (NEC)-IECs that challenged by IL-1β [64].

Moreover, ω-3 PUFA conferred antimicrobial activity against the common feed contaminants such as the mycotoxin deoxynivalenol (DON) and bacterial lipopolysaccharides (LPS). Accordingly, ω-3 PUFA (EPA or DHA) pre-treatment of porcine IPEC-1 cells reversed the DON-induced damage on cell proliferation, viability, tight junction proteins (CLDN-1, ZO-1), and the monolayer integrity (TEER) [65]. Also, the DON-induced cell membrane damage was inhibited by ω-3 PUFA as indicated by marked reduced in the cytosolic lactate dehydrogenase (LDH) release into the cell culture medium. Additionally in the same study, ω-3 PUFA reduced the number of necrotic cells and the expression of proteins that involved in DON-induced apoptosis or necroptosis signaling such as the caspase-3/-7, TNF receptor-1 (TNFR1), receptor-interacting protein kinase (RIPK)-1/-3, phosphorylated mixed lineage kinase-like protein (MLKL), phosphoglycerate mutase family 5 (PGAM5), dynamin-related protein 1 (DRP1) and high mobility group box-1 (HMBG1) [65]. Similarly, in our previous study, ω-3 PUFA (EPA and DHA) treatment significantly enhanced the proliferation of porcine IPEC-J2 cells. It further inhibited the apoptosis (caspase-3/-7) and secured the cell viability or cell membrane integrity (LDH release) that was damaged by different biological and chemical stressors as LPS, DSS, and H_2_O_2_ [66].

##### In vivo studies

Previously ω-3 PUFA secured the intestinal barrier and decreased the disease activity in both human and animal models of IBD (Tables 1 and 2). At the intestinal level, disease activity was assessed by various parameters not limited to the expression of pro-inflammatory mediators, intestinal weight/length ratio, villus height/crypt depth (V/C) ratio, inflammatory cell infiltration, epithelial and mucosal morphology. Accordingly, in rat acetic acid-colitis, ω-3 PUFA reduced the expression of pro-inflammatory eicosanoids (PGE2, LTB4, TXB2), macrophage infiltration, mucosal, and tissue damage [68]. Following this, ALA supplementation in TNBS-colitis rats inhibited the protein expression of intercellular adhesion molecule-1 (ICAM-1), vascular cell adhesion molecule (VCAM-1), and vascular endothelial growth factor receptor-2 (VEGFR-2) that mediates leukocyte recruitment and angiogenesis in IBD [69].

**Table 2:** Clinical impacts of omega-3 polyunsaturated fatty acids in the patients with inflammatory bowel disease

Clinical study	Under IBD medications	ω-3 PUFA(s) assessed	Immune response and morphological changes	Reference
Ethanol-induced duodenum lesions	No	Fish oil	↑LTC5; ↓endoscopic and histologic lesions; =(PGE2, PGI2, TXB2)	[87]
Pediatric ulcerative colitis in remission	Yes	EPA	↓LTB4; =histological score	[88]
Active distal proctocolitis	No	Fish oil	↑Clinical, endoscopic and histological remission	[89]
Active ulcerative colitis	No	Fish oil	↑Clinical and histological remission	[90]
Active ulcerative colitis	No	Fish oil	↑Endoscopic remission; =clinical and histological score	[91]
Active ulcerative colitis	No	Fish oil	↑Clinical, endoscopic and histological remission	[92]
Active ulcerative colitis or CD	Yes	Fish oil	↑Clinical, endoscopic and histological remission	[93]
Active ulcerative colitis or CD	Yes	Fish oil	↓(PGE2, PGI2, TXB2)	[94]
Active ulcerative colitis	Yes	Salmon fillet	↑Clinical, endoscopic and histological remission	[95]
Active ulcerative colitis	Yes	EPA	↑Clinical and endoscopic remission	[96]
Ulcerative colitis in remission	No	Fish oil	↑Temporary clinical, macroscopic and histologic remission; delayed early relapse	[97]
Ulcerative colitis in remission	Yes	EPA	↑Endoscopic and histological remission	[98]
Ulcerative colitis in remission	Yes	EPA	↑(*IL-10, SOCS3*, *IL-22*, HES-1, KLF-4, goblet cell abundance, endoscopic and histological remission); ↓phospho-STAT3; =(IL-10, SOCS3, *STAT3*, Ki67, *c-MYC*, *LGR5, MUC2, HES-1, KLF-4*)	[99]
Active ulcerative colitis or in remission	Yes	Fish oil	=(Bleeding, disease relapse, endoscopic and histological score)	[100]
Active ulcerative colitis	Yes	Fish oil	=Endoscopic and histologic scores	[101]
Quiescent ulcerative colitis	Yes	EPA and DHA	=(Disease relapse, endoscopic and histological score)	[102]

The arrow indicates an increase (↑) or decrease (↓) in the level or activity of the different parameters analysed, “=” symbol designates unchanged parameters. *CD* Crohn’s disease, *COX* Cyclooxygenase, *c-MYC* c-Myelocytomatosis proto-oncogene, *HES-1* Hairy and enhancer of split-1, *IBD* Inflammatory bowel disease, *IL* Interleukin, *Ki67* Cell proliferation marker, *KLF-4* Kruppel-like factor-4, *LGR5* Leucine-rich repeat-containing G-protein coupled receptor 5, *LT* Leukotriene, *PG* Prostaglandin, *PUFA* Polyunsaturated fatty acid, *p-STAT3* Phosphorylated signal transducer and activator of transcription 3, *SOCS3* Suppressor of cytokine signalling-3, *STAT3* Signal transducer and activator of transcription-3, *TXB* Thromboxane

Further, fish oil treatment supported the recovery of TNBS- or DSS-induced colitis in murine models. Accordingly, fish oil reduced the gene or protein expression of pro-inflammatory eicosanoids (PGE2, COX-2, LTB4 or LTC4) and the molecules of cytokine signaling (TNF-α, IL-6 or NF-κB) [70, 71]. The level of disease-specific enzymatic markers such as iNOS, alkaline phosphatase and, myeloperoxidase (MPO) were also suppressed [71]. Additionally, macroscopic injury of the intestinal mucosa (ulceration, necrosis, inflammation) [70, 71], inflammatory cell infiltration [72], colon wall thickness, and, the weight/length ratio [72] were significantly reduced. Moreover, fish oil restituted the goblet cell abundance [71], glutathione level [71], mucosal, and tissue integrity [70, 71].

For the first time, DHA-derived MaR1 and EPA-derived RvE1 were shown to suppress the disease activity in murine colitis. Accordingly, MaR1 supplementation in DSS-colitis rats increased the expressions of different tight junction proteins (ZO-1, OCLN), while inhibited the mucosal damage, colon shortening, inflammatory cell infiltration, and expressions of different pro-inflammatory mediators (PGE2, MPO, ROS, TNF-α, IL-1β/-6) [72]. Importantly, MaR1 inhibited the protein expression of TLR4/NF-κB signaling (TLR4, p-NF-κB-p65) and instead activated the anti-inflammatory nuclear factor erythroid 2-related factor 2 (NRF2)/heme oxygenase-1 (HO-1) signaling [72]. Similarly, MaR1 intervention ameliorated the severity of acute and chronic DSS-colitis in mice by reducing epithelial damage, inflammatory cell infiltration, hyperaemia, colon wall thickness, colon shortening, and expression of pro-inflammatory genes or proteins (MPO, NF-κB, IL-1β, IL-6, TNF-α, interferon[INF]γ, *ICAM-1*) [75]. Likewise, RvE1 facilitated tissue repair in peritonitis mice with TNBS-colitis, while significantly reduced the leukocyte infiltration, MPO activity, and the expression of genes corresponding to pro-inflammatory response (*COX-2, TNF-α, IL-12p40, iNOS*) [76]. Generally, a steep oxygen gradient exists in the intestinal barrier to support the sustenance of gut microbiota and other barrier functions. An imbalance in the gradient can instigate barrier degenerative diseases such as ischemic-reperfusion and NEC [103]. Earlier, ω-3 PUFA remediated the experimental NEC induced by hypoxia, formula feeding, or its combination in murine models. Accordingly, DHA intervention significantly decreased tissue necrosis, incidence of disease, and the gene expression of pro-inflammatory mediators such as *TLR2/4* and platelet-activating factor receptor (*PAFR*) in the NEC-rat pups [73]. Moreover, ω-3 PUFA (EPA or DHA) administration in maternal rats during gestation, significantly accumulated in the intestinal phospholipids of prematurely delivered rat pups. This subsequently ameliorated the impact of experimental NEC induced in rat pups by inhibited the gene expression of pro-inflammatory *IκBα/β* and instead activated the anti-inflammatory *PPARγ* gene. Besides, the gene expression of PGE2 receptor *EP3* and PGD2 receptor *DP2* was increased, while the mucosal damage, inflammatory cell infiltration, and incidence of disease was decreased [74].

As observed in cellular studies, ω-3 PUFA supported the recovery from bacterial infections in murine models. Specifically, fish oil administration in mice significantly reduced the *Citrobacter rodentium (C. rodentium*)*-*induced mucosal damage, inflammatory cell infiltration, enterocyte proliferation, apoptosis, and intestinal permeability to FD4. Also, fish oil increased the gene expression of anti-inflammatory cytokine *IL-10*, while suppressed the gene expressions of pro-inflammatory cytokines and chemokines such as *IL-6, IL-17A, IFNγ,* monocyte chemoattractant protein-1 (*MCP-1*), keratinocyte cytokine, and macrophage inflammatory protein-2 *(MIP-2)* [77]. Further, ω-3 PUFA (EPA, DHA or fish oil) intervention in mice, attenuated the expression of genes or proteins involved in LPS-activated TLR4/NF-κB signaling (*TLR4, MyD88*, NF-κB-p65*, NF-κB* or transforming growth factor-β-activated kinase [TAK]1) and the downstream pro-inflammatory response (*TNF-α, IL-1β/-6, INFγ* or COX-2) [78, 79]. Besides, the expression of tight junction proteins (E-cadherin, ZO-1, OCLN), ω-3 PUFA receptor gene (G-protein coupled receptor *[GPR]*120), and tissue repair were significantly enhanced. The gene expression of anti-inflammatory cytokine *IL-10, MUC2*, and its regulatory gene, free fatty acid receptor-2 (*FFAR-2*) were also substantially increased [79].

Apart from infections, weaning imparts tremendous inflammatory and metabolic stress in pigs due to changes in gastrointestinal physiology [104]. Previously, administration of fish oil in pigs significantly downregulated the protein expression of pro-inflammatory eicosanoids (PGE, PGI2, TXB2) through ω-3 PUFA enrichment in the intestinal phospholipids. Although the morphology of colonic mucosa remained stable, the crypt depth was significantly reduced [80] (Table 1). Further, fish oil administration during the course of gestation to lactation in sows markedly increased the ω-3 PUFA levels in ileal phospholipids. The maternal diet subsequently instigated an age-dependent decrease in villus height (d 0), crypt depth (d 7), and HRP permeability (d 0–28) in the piglet ileum. These effects were reported to prevent the intestinal pathologies associated with IEL functioning during the neonatal period [81].

In similar maternal physiology, supplementation of extruded linseed oil temporarily increased the FD4/LPS permeability in the piglet jejunum explants until d 28 and thereafter decreased at d 52 [82]. The distinct outcome of these two trials could have possibly aroused from a difference in linseed oil formulation administered, dose-effect, or animal physiology. Nevertheless, the influence of maternal dietary ω-3 PUFA on piglet intestinal development and health status is unclear and hence needs further investigations. In another study, fish oil administration in weaned piglets protected from LPS-induced damage on the intestinal barrier by enhancing the expressions of tight junction proteins (OCLN, CLDN-1), villus height, and V/C ratio [83]. Besides, the expression of genes corresponding to the TLR4 pathway such as the *TLR4,* myeloid differentiation primary response 88 **(***MyD88*), interleukin-1 receptor-associated kinase-1 (*IRAK1*), and TNFR-associated factor-6 (*TRAF6*) were suppressed. The expression of inflammatory genes as *NOD2* and *RIPK-2*, and the expression of proteins as diamine oxidase, caspase-3, and heat shock protein-70 (HSP70) were also significantly downregulated [83]. Furthermore, fish oil supported the remission of DSS-colitis in the early-weaned piglets by enhancing enterocyte mitosis and gene expression of PPARγ-responsive uncoupling protein-3 *(UPC3)* [84]. Moreover, EPA intervention in piglets moderately supported the recovery of ileum ischemic injury by decreasing the expression of PGE2 protein and barrier flux to H^3^-mannitol/C^14^-inulin, while increased the expression of *COX-2* gene and TEER values [85]. In another study that administered fish oil in pigs, exhibited a significant reduction in LPS permeability across the ileal explants [67].

**Fig. 3:**
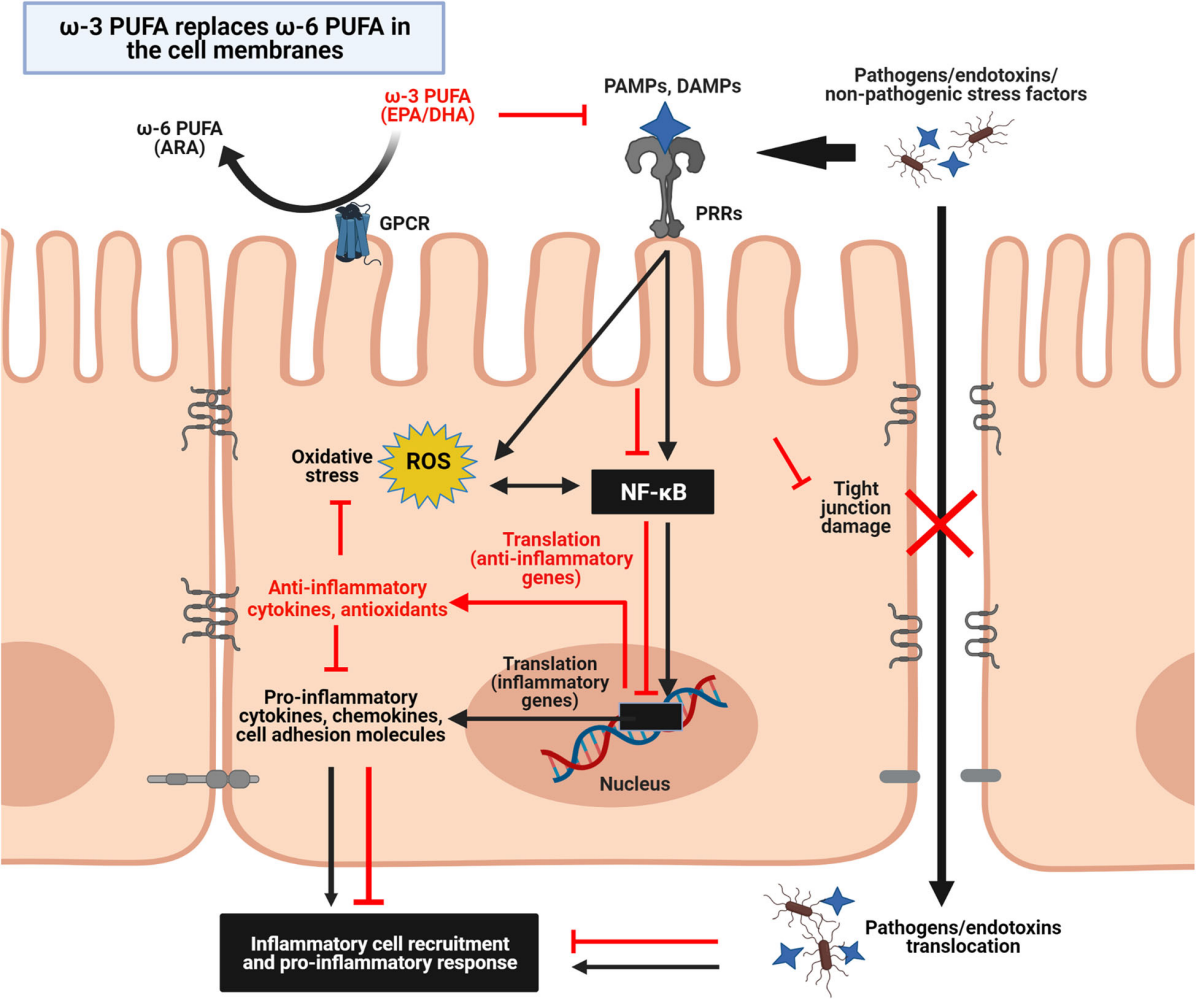
Model summarizing the immunomodulatory mechanisms of omega-3 polyunsaturated fatty acids in the intestinal epithelium. In arachidonic acid (ARA)-enriched cell membranes, during an infection or injury, pathogen-associated molecular patterns (PAMPs) or damage-associated molecular patterns (DAMPs) binds with the host-specific pattern-recognizing receptors (PRRs) and activate the nuclear factor-κB (NF-κB) signaling to release pro-inflammatory cytokines, chemokines and reactive oxygen species (ROS). Subsequently, these mediators recruit the inflammatory cells from lamina propria and exert a strong pro-inflammatory response. Pro-inflammation also damages the integrity of the epithelial barrier by disrupting the tight junction proteins. Loss of epithelial integrity aggravates inflammation by facilitating the translocation of luminal pathogens and endotoxins into the circulatory system (Black lines). Dietary supplementation of eicosapentaenoic acid (EPA) and docosahexaenoic acid (DHA) ameliorates the pro-inflammatory response by replacing ARA in specific cell membrane G-protein-coupled receptors (GPCR) and instead stimulates the production of anti-inflammatory cytokines and antioxidants (Red lines). For references, see text. Figure created using BioRender.com

In clinical settings, ω-3 PUFAs are administered either individually or as an immunological adjuvant for controlling different IBDs including Crohn’s disease (CD), ulcerative colitis, and distal proctocolitis (Table 2). Accordingly, in subjects with ethanol-induced duodenal injury, fish oil administration relieved macroscopic lesions by increased the protein expression of anti-inflammatory eicosanoid, LTC5 [87]. Also, EPA suppressed the mucosal expression of pro-inflammatory eicosanoid as LTB4 in pediatric ulcerative colitis under remission [88]. Further, administration of fish oil in patients with active ulcerative colitis or distal proctocolitis, witnessed a marked reduction in disease activity as observed from improvements in clinical, endoscopic, and histological scores [89–92]. In active ulcerative colitis or CD patients undertaking IBD medications, ω-3 PUFA (fish, fish oil or EPA) administration reduced the disease activity [93, 95, 96] and protein expression of pro-inflammatory eicosanoids (PGE2, PGI2, TXB2) [94] by incorporating in the mucosal phospholipids [93–95]. Similarly, the health status of ulcerative colitis patients in remission with or without IBD medication was improved by fish oil or EPA administration [97–99]. Specifically, the disease activity and mucosal inflammation were remediated by activating the gene expression of anti-inflammatory mediators such as the *IL-10*, and suppressor of cytokine signaling-3 (*SOCS3*), while reduced the protein expression of phosphorylated signal transducer and activator of transcription 3 (p-STAT3). Additionally, the abundance of goblet cells, *IL-22*, and proteins that modulate the secretory and absorptive cell lineage such as the hairy and enhancer of split-1 (HES-1) and kruppel-like factor-4 (KLF-4) were also increased [99]. On the contrary, few studies have reported that ω-3 PUFA (fish oil, EPA, or DHA) had no beneficial effects in the patients with either active, quiescent, or remitted ulcerative colitis under IBD medications [100–102]. Various factors could have contributed to the differential outcome of ω-3 PUFA diet in patients such as the age, metabolic status, dose, or the impact of co-administered IBD medications. A schematic representation summarizing the anti-inflammatory and antioxidative mechanisms of ω-3 PUFA through NF-κB inhibition in IEL is reported in Fig. 3. Table 1 and 2.

### Citrus pectin

#### Structure and molecular mechanism

**Fig. 4:**
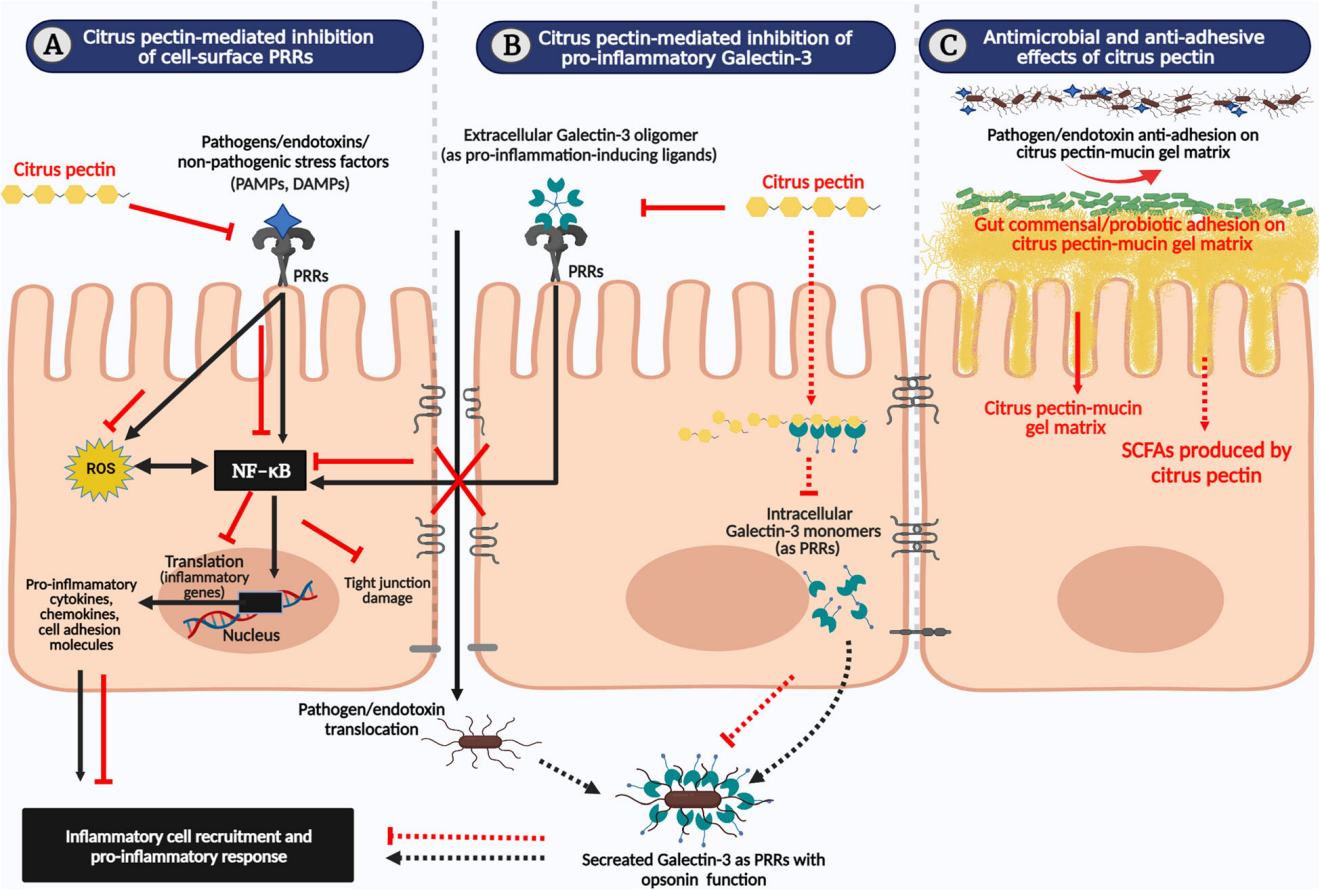
Model summarizing the immunomodulatory and antimicrobial mechanisms of citrus pectin in the intestinal epithelium. (A) Pathogen-associated molecular patterns (PAMPs) or damage-associated molecular patterns (DAMPs) activate the nuclear factor-κB (NF-κB)-mediated pro-inflammatory response and damages the epithelial integrity as described in Fig. 3. (Black solid lines). Dietary citrus pectin (CPn) blocks the cell surface pattern-recognizing receptors (PRRs) and prevents the PAMPs or DAMPs from activating NF-κB (Red solid lines). (B) During infection or injury, extracellular Galectin-3 (Gal-3) acts as ligands to cell-surface PRRs and activates NF-κB-mediated pro-inflammatory response (Black solid lines). CPn can modify the cell-surface PRRs and prevent the Gal-3 from binding (Red solid lines). Alternatively, cells secrete intracellular Gal-3 that acts as PRRs to pathogens or endotoxins and recruits immune cells enabling opsonization (Black dotted lines). CPn can directly bind the intracellular Gal-3 and block its opsonin function (Red dotted lines). (C) CPn binds with mucin glycoproteins and forms gel-matrix that selectively support the adhesion of probiotics and gut commensals, while repel the pathogens (Red solid lines). Alternatively, CPn directly interacts with the pathogen and inhibits its growth or indirectly give rise to short-chain fatty acids (SCFAs) that protects barrier health (Red dotted lines). Abbreviations: ROS, Reactive oxygen species. For references, see text. Figure created using BioRender.com

CPn is a highly branched, complex heteropolysaccharide derived from the peels and pomace of citrus fruits. The structure of CPn involves a covalently linked, galacturonic acid backbone with three major polysaccharide units of homogalacturonan, rhamnogalacturonan, and substituted galacturonans [105, 106]. Generally, the small intestine cannot assimilate native pectins due to its large molecular weight (60–300 kDa), complex structure, and higher degrees of esterification (DE ~ 70%). However, pH or enzymatic treatment yields compounds of low molecular weight (15 kDa) and reduced DE (< 5%). The modified CPn exhibit enhanced intestinal absorption and transportation [107, 108]. Interest in utilizing CPn as a potential feed additive is emerging from the recent pieces of evidence on its anti-inflammatory, antimicrobial, and prebiotic activities [30, 34, 109, 110]. Owing to the complex physiochemical structure of CPn, its precise molecular mechanism is fairly established [34]. However, previous reports on its ability to interact with TLRs, Galectin-3 (Gal-3) or produce short-chain fatty acids (SCFAs) could be attributed to part of its immunomodulatory mechanisms in the intestinal barrier [30, 98] [Fig. 4]. Interestingly, non-digestible carbohydrates and LPS share a structural similarity with respect to carbohydrate-containing regions that interact with IECs and other immune cells [98]. CPn belongs to the category of non-digestible carbohydrates and was shown to interact with TLR2/4 that resulted in modulating NF-κB in both IECs and immune cells [30, 98, 100–102, 105–114]. Another mechanism by which CPn controls inflammation is by inactivating Gal-3 signaling proteins. Gal-3 belongs to the lectin family, a class of carbohydrate-binding proteins that dispersed in both intracellular and extracellular regions. It possesses a unique carbohydrate-recognition domain (CRD) that binds to the β-galactoside sugars [106, 115]. Gal-3 was reported to involve in the pathogenesis of microbial infections, cancer, and several other inflammatory diseases [116, 117]. The mechanism by which Gal-3 regulates inflammatory response is multifaceted. In intestine, it stimulates pro-inflammatory responses by acting as ligands to TLRs of IECs [117, 118] [Fig. 4B]. Previously, in rat pups, induction of experimental NEC was shown to activate Gal-3-mediated TLR4/NF-κB signaling [118]. Alternatively, Gal-3 act as PRRs to pathogens or LPS and recruit immune cells for opsonization [117] [Fig. 4B]. Of note, orally or intravenously administered CPn are able to cross the intestinal barrier and inhibit Gal-3-mediated inflammation by binding its CRD region [119, 120] [Fig. 4B]. Currently, the application of CPn as Gal-3 inhibitor is emerging as a novel strategy for treating cancer and many inflammatory diseases [35, 106, 108, 121]. From all these observations, it could be possible that certain carbohydrate regions of CPn, LPS, and Gal-3 that binds to IECs or immune cells are quite similar. This could have enabled CPn to prevent inflammation by competing with LPS or Gal-3 for the TLR-binding region [Fig. 4A, B]. Moreover, the SCFAs produced during the fermentation of dietary fibers such as CPn was reported to improve the intestinal barrier functions [98].

#### Impact on intestinal barrier under normal and stress conditions

##### In vitro studies

CPn self-assembles into dense hydrogel matrix on contact with the mucin glycoproteins of IEL [122] [Fig. 4C]. The gel-forming ability of CPn (DE94% and DE25%) was demonstrated using the commercially available mucins and on the mucosal surface of porcine colonic tissues. In this study, CPn-DE94% formed a gel matrix at the tissue surface, while CPn-DE25% permeated deeper towards the tissue wall. Besides, the net electrical charge of CPn influenced the rheological strength of the gel [123]. Following, different oligosaccharides of CPn were shown to cross the Caco-2 cell monolayer based on the degree of polymerization in a transwell culture system. Particularly, only the short-chain galectins and arabinogalactans could transverse, but not the galacturonic acid [107].

A recent study demonstrated the immunomodulatory behaviour of in vitro digested citrus pulp on the intestinal barrier using IPEC-J2 cell model. Specifically, it inhibited the gene expressions of *TLR4* and *CLDN-1* and instead activated *NOD1* under a stress-free environment [124] (Table 3). Further, many studies demonstrated the barrier protective properties of CPn under inflammatory conditions. Accordingly, CPn with different degrees of methyl esters (DM32%, DM59%, and DM64%) secured the integrity (TEER) and reduced the permeability (lucifer yellow flux) of mouse CMT93 cell monolayer that was disrupted by the pathogenic challenge of *C. rodentium*. Interestingly, using a reporter cell assay, CPn was shown to activate the NF-κB/AP-1 signaling via TLR2 in the CMT93 cells independent of *C. rodentium* challenge. Additionally, CPn imparted anti-adhesive effects on *C. rodentium* by interacting with the pathogen instead of CMT93 cells [113]. Likewise, both native and enzymatically modified citrus residues prevented the Caco-2 cells from secreting IL-8 upon the pathogenic challenge of *Salmonella typhimurium (S. typhimurium)* and *Listeria monocytogenes (L. monocytogenes)* [33]. In this study, citrus treatment selectively supported the cellular adhesion of probiotics *(Lacticaseibacillus casei* and *Bifidobacterium lactis)*, while repelled the pathogens (*S. typhimurium* and *L. monocytogenes)* [Fig. 4C]. Moreover, the enzymatically modified citrus residues exhibited enhanced antibacterial and prebiotic activities in comparison to the unmodified [33]. In another study, CPn (DM30%, DM56%, and DM74%) secured the integrity (TEER) of T84 cell monolayer from the barrier disrupting agent such as the phorbol esters [114].

**Table 3:** Effects of citrus pectin in the intestinal cell and animal models

Model of study	Stress by	CPn(s) assessed	Immune response and morphological changes	Reference
**In vitro**				
Human T84 cells	Phorbol esters	CPn (DM30%, DM56%, DM74%)	↑TEER	[114]
Human Caco-2 cells	*Salmonella typhimurium* (pathogen), *Listeria monocytogenes* (pathogen), *Lacticaseibacillus casei* (probiotic), *Bifidobacterium lactis* (probiotic)	CPn or citrus residues after juice/pectin extraction	↓(IL-8, pathogen adhesion and invasion); ↑probiotic adhesion	[33]
Mouse CMT93 cells	*Citrobacter rodentium*	CPn (DM32%, DM59%, DM64%)	↓(Pathogen adhesion, lucifer yellow flux); ↑TEER	[113]
Porcine IPEC-J2 cells	Non-stimulated	Fermented citrus pulp	*↓(TLR4, CLDN-1); ↑NOD1*	[124]
**In vivo**				
Rats	Non-stimulated	CPn	↑(Ki67^+^ cells, intestinal length and weight, cecum SCFAs, mucosal wet weight, protein and DNA content)	[125]
Rats	Methotrexate-colitis	CPn	↓(Organ water content, MPO, intestinal permeability, bacterial translocation); ↑(mucosal protein, DNA and RNA content)	[126]
Mice	Acetic acid-colitis	CPn	↓(ROS, MPO, granulocyte adhesion, colon damage)	[127]
Mice	Doxorubicin-ileitis	CPn (DM7%)	↓(TNF-α, MCP-1, CXCL1, IL-6, inflammatory cell infiltration, crypt cell apoptosis); =(IL-10, cecum SCFAs)	[32]
Mice	DSS-colitis	CPn or citrus residues after juice extraction	*↓(TNF-α, IL-1β, IL-16, iNOS, ICAM-1,* colon weight/length ratio); *↑(MUC3, ZO-1, OCLN)*	[128]
Mice	DSS-colitis	CPn (DE68.01 ± 0.43%, DE41.61 ± 0.12%, DE38.09 ± 0.78%)	↓(IL-6, IL-17, MPO, FD4/LPS flux, epithelial erosion, ulceration, inflammatory cell infiltration, colon weight/length ratio); ↑(ZO-1, goblet cell abundance, crypt and villus structure); =OCLN	[31]
Mice	DSS-colitis	CPn, CPn methanol extracts or methanol residues	↓(*TNF-α, IL-1β, IL-6, CXCL2, IL-17a*, ulceration, erosion, inflammatory cell infiltration, colon shortening); ↑(ZO-2, OCLN, CLDN-3/-7, JAM-A)	[129]
Mice	DSS-colitis	Methanol extracted CPn	↓(*IL-6, MCP-1, CXCL2,* epithelial damage, inflammatory cell infiltration, colon shortening); ↑(ZO-1/-2, CLDN-3/-7, crypt structure, goblet cell abundance); =*IL-17a*	[130]
Mice	DSS-colitis	CPn	↓(TNF-α, IL-12, colon shortening)	[131]
Cats	Indomethacin-small intestinal lesions	CPn	↓Mucosal ulceration and lesions	[132]
Chicken	*Eimeria maxima* coccidiosis	CPn	↓(*IL-12β*, serosa thickness, schizont count in enterocytes); ↑(*IFN-γ, IL-1β*, goblet cell abundance, V/C ratio, cecum weight, cecum SCFAs); =*(MUC2, IL-8)*	[133]
Weaned piglets	Non-stimulated	Citrus pulp	=*(IL-6, IL-1β, TNF-α, IFN-γ, IL-10, SOCS3)*	[134]

The arrow indicates an increase (↑) or decrease (↓) in the level or activity of the different parameters analysed, “=” symbol designates unchanged parameters. *CLDN* Claudin, *CPn* Citrus pectin, *CXCL* Chemokine C-X-C motif ligand, *DE* Degree of esterification, *DM* Degrees of methyl esterification, DSS Dextran sulphate sodium, *FD4* Fluorescein isothiocyanate-labelled dextran 4 kDa, *ICAM-1* Intercellular adhesion molecule-1, *IFNγ* Interferon γ, *IL* Interleukin, *iNOS* Inducible nitric oxide synthase, *JAM* Junctional adhesion molecule, *Ki67* Cell proliferation marker, *LPS* Lipopolysaccharides, *MCP-1* Monocyte chemotactic protein-1, *MPO* Myeloperoxidase, *MUC* Mucin, *NF-κB* Nuclear factor-κB, *OCLN* Occludin, *ROS* Reactive oxygen species, *SCFAs* Short-chain fatty acids, *SOCS3* Suppressor of cytokine signalling-3, *TEER* Transepithelial electrical resistance, *TLR* Toll-like receptor, *TNBS* 2,4,6-Trinitrobenzene sulfonic acid, *TNF* Tumour necrosis factor, *ZO* Zonula occludens

##### In vivo studies

Previously, CPn administration in rats under normal physiology, significantly increased the mucosal proliferation (Ki67^+^ cells), intestinal length, weight, and the level of cecum SCFAs [125](Table 3). In agreement with cellular studies, CPn potentially ameliorated the intestinal barrier inflammation under different stress conditions in animal models. Accordingly, in rats with methotrexate-colitis, supplementation of CPn reduced the intestinal barrier permeability, bacterial translocation, MPO expression, and intestinal water content. It further stimulated mucosal replenishment as indicated by an increase in mucosal wet weight, protein, and nucleic acid content [126]. In the mice with acetic acid-colitis, CPn reduced the peritoneal granulocyte adhesion, intestinal tissue injury, ROS production, and MPO expression [127]. Similarly, in the mice with doxorubicin-induced ileitis, CPn-DM7% administration substantially reduced the crypt cell apoptosis, inflammatory cell infiltration, and protein expression of pro-inflammatory cytokines and chemokines (TNF-α, MCP-1, IL-6, chemokine C-X-C motif ligand *[CXCL]-*1) [32]. In the DSS-colitis mice, supplementation of CPn or citrus residues (from juice extraction) decreased the expression of pro-inflammatory genes (*TNF-α, IL-1β/-16, iNOS*, *ICAM-1*) and colon weight/length ratio. Additionally, CPn restituted the intestinal barrier integrity as observed from increased expression levels of *MUC3* and tight junction proteins *(ZO-1, OCLN)* [128]. In a similar disease model of mice, dietary CPn (DE68.01 ± 0.43%, DE41.61 ± 0.12%, and DE38.09 ± 0.78%) reduced the expression of pro-inflammatory proteins (IL-6/-17, MPO), intestinal permeability (FD4/LPS flux), inflammatory cell infiltration, colon weight/length ratio, epithelial and mucosal damage. Additionally, CPn increased the goblet cell abundance, ZO-1 expression, and V/C architecture [31].

In another study, administration of CPn, its methanol extracts, or methanol residues in the DSS-colitis mice, attenuated the mucosal damage, inflammatory cell infiltration, colon shortening, and gene or protein expression of pro-inflammation cytokines and chemokines (*TNF-α, IL-1β, IL-6, IL-17a,* IL-12, *MCP-1 or CXCL2*) [129–131]. As observed earlier, CPn supported the recovery of barrier integrity by improving the goblet cell abundance [31, 129] and the expression of different tight junction proteins (ZO-1/-2, OCLN, CLDN-3/-7, or JAM-A) [129, 130]. In cats, CPn ameliorated the small intestinal ulceration and lesions commonly caused by the side effects of NSAIDs as the indomethacin [132]. In chicken coccidiosis, CPn administration decreased the serosa thickness, number of schizonts in the ileal enterocytes, and the gene expression of pro-inflammatory cytokine *IL-12β*. In addition to improvement in goblet cell abundance, V/C ratio, cecum weight, levels of SCFAs, and the gene expression of pro-inflammatory cytokines *(INFγ, IL-1β)* were also increased. Although CPn displayed beneficial effects to a certain extent, on the contrary, it also activated inflammatory response and decreased the growth performance of un-infected birds [133]. Following this, supplementation of citrus pulp in weaned piglets did not alter the intestinal morphology or inflammatory status [134]. In some of the in vivo trials, CPn administration significantly enhanced the level of the cecum or fecal SCFAs, which could have contributed to the changes observed in intestinal morphology and its immune status [129, 130, 133].

### Milk exosomes

#### Structure and molecular mechanism

**Fig. 5:**
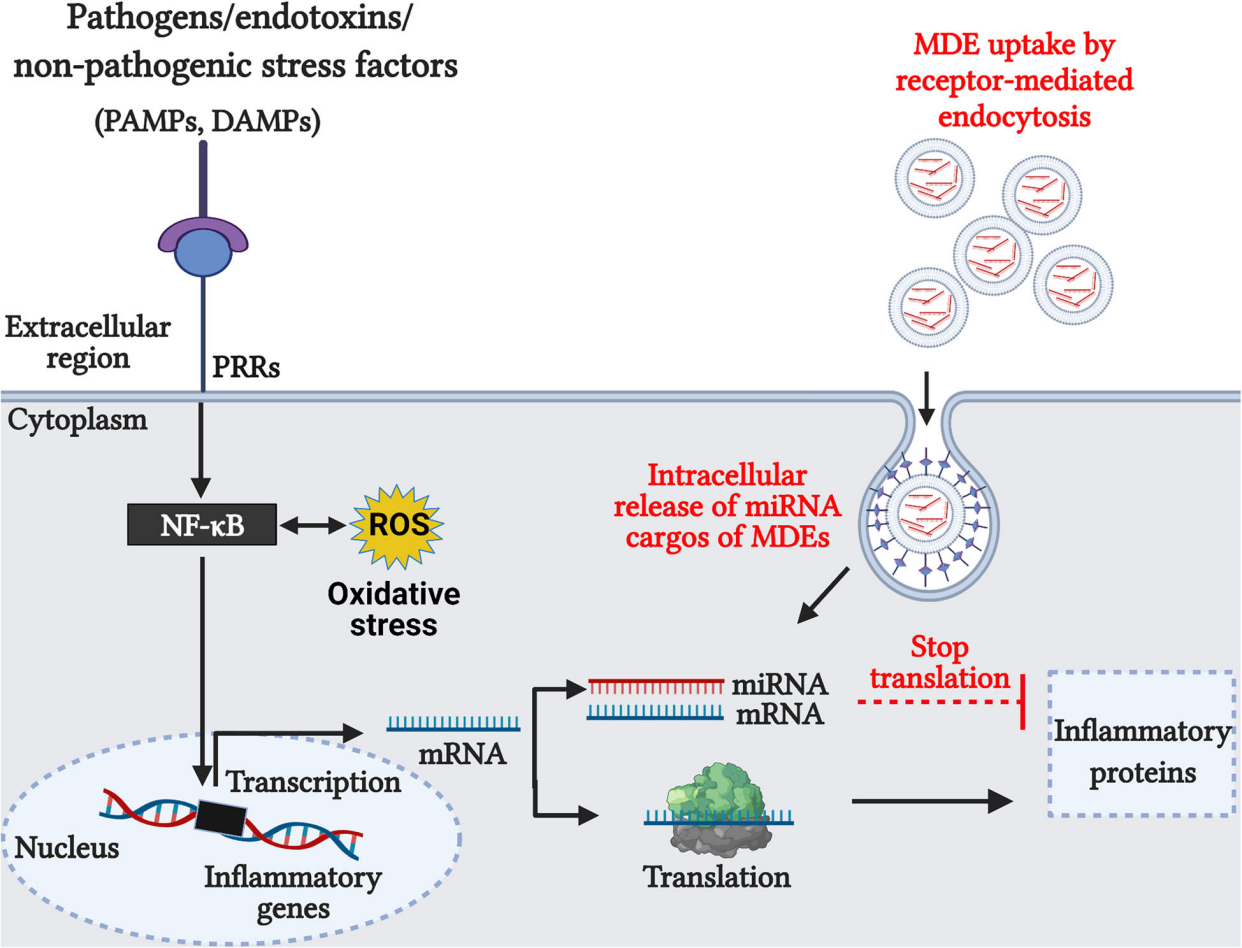
Model summarizing the immunomodulatory mechanisms of milk-derived exosomes in the intestinal epithelium. Host cells when sensed pathogen-associated molecular patterns (PAMPs) or damage-associated molecular patterns (DAMPs) via pattern-recognizing receptors (PRRs), activate the nuclear factor-κB (NF-κB)-mediated pro-inflammatory response, oxidative stress, and cell-death pathway (Black lines). Dietary milk-derived exosomes (MDEs) deliver their miRNA/peptide cargo to the intestinal epithelial cells via receptor-mediated endocytosis. Subsequently, the MDE-miRNAs bind to the complementary mRNAs in the cells and inhibit the synthesis of proteins specific for NF-κB signaling (Red lines). Abbreviations: ROS, Reactive oxygen species; miRNA, microRNA; mRNA, messenger RNA. For references, see text. Figure created using BioRender.com

Exosomes are membrane-bound, extracellular vesicles of 30–150 nm size and 1.13–1.19 g/mL density. It carries cargos of nucleic acids, proteins, lipids, and other signaling molecules for specific cellular functions [135]. Exosomes are either directly released from the cell membranes or produced within the cells and then externally released [136]. Most cells secrete exosomes into various body fluids including the blood and milk [136, 137]. Recent bioinformatical analysis has identified the presence of numerous microRNAs (miRNAs) and peptides in the milk-derived exosomes (MDEs) relevant for developmental and immune-related activities of the intestinal barrier [38, 39, 138]. Generally, miRNAs are single-stranded, non-coding RNA molecules of 18–25 nucleotides long. The primary miRNA is synthesized in the nucleus and then transported to the cytoplasm, where it is processed into mature miRNA. In association with the RNA-inducing silencing complex, the mature miRNA binds to a complementary mRNA and inhibit protein synthesis [139]. Through dietary MDEs, the molecules that are involved in initiation, propagation, and resolution phases of intestinal inflammation can be controlled [Fig.5].

#### Impact on intestinal barrier under normal and stress conditions

##### In vitro studies

Dietary MDEs survives the intestinal digestion and are subsequently taken up by the IECs or transported across [140–142]. The miRNA sequencing of MDEs has revealed that even harsh gastric/pancreatic digestion had a limited impact on its miRNA profiles [140, 141]. Further, MDE uptake is driven by receptor-mediated endocytosis in various cell types including the small intestinal IECs [143]. This shows how the dietary MDEs stably cross the intestinal barrier and enter systemic circulation in order to establish specific ‘cell-to-cell’ communication. Moreover, maternal MDEs carry miRNAs, proteins, and other growth-promoting factors necessary for the maturation of infant gut immunity [144]. Several studies have demonstrated the ability of miRNAs to control the developmental and immune-related activities of the intestinal barrier (Table 4).

**Table 4:** Effects of milk-derived exosomes in the intestinal cell and animal models

Model of study	Stress by	MDEs/MDE-miRNAs from	Immune response and morphological changes	Reference
**In vitro**				
Porcine IPEC-J2 cells	Non-stimulated	Porcine milk	↑(Cell proliferation, *CDX2, IGF-1R, PCNA*, miRNAs targeting FAS, SERPINE and p53 pathways); ↓(*FAS, SERPINE*, *p53*)	[138]
Rat IEC-18 cells	Non-stimulated	Rat milk	↑(Cell viability, proliferation, *PCNA*, *LGR5*)	[145]
Human CCD841 cells or human LS123 cells	Non-stimulated	Human milk	↑(Cell proliferation, collagen type-I); ↓(*twist1*, PTEN) in CCD841 cells; = LS123 cells	[146]
Human LS174T cells	Non-stimulated	Bovine milk	↑(Mucin secretion, *TFF3*, *MUC2, GRP94*)	[147]
Human FHC cells	Mechanical wound	Human term or preterm milk	↑(Cell proliferation, migration and wound healing)	[38]
Porcine IPEC-J2 cells	LPS	Porcine milk	↑(Cell viability, IκBα); ↓(*TLR4, MyD88,* p-IκBα, p-NF-κB-p65, p-NF-κB, NF-κB nuclear translocation*, Tp53, FAS, Caspase-3*, IL-1β, IL-6, TNF-α)	[148]
Porcine IPEC-J2 cells	LPS	Porcine milk	↑(Cell viability, *IκBα*); ↓(*TLR4*, p-IκBα, p-NF-κB-p65, p-NF-κB, *NF-κB*, *Tp53, p53, FAS*, *Caspase-3,* IL-1β, IL-6, TNF-α)	[149]
Porcine IPEC-J2 cells	DON	Porcine milk	↑(Cell viability, proliferation, *β-catenin, CCND1*, *Akt, ZO-1, OCLN, CLDN1, PCNA*, miRNAs targeting p53 pathway); *↓(Tp53, FAS, SERPINE1, p21)*	[150]
Rat IEC-6 cells	Hypoxia	Yak milk	↑(Cell viability, proliferation, PHD-1); ↓(HIF-1α, VEGF, p53)	[151]
Rat IEC-6 cells	Hypoxia	Yak milk	↑(Cell viability, Ki67^+^ cells, PHD-1); ↓(HIF-α, VEGFA, p53, Bax, caspase-3/-9)	[39]
Rat IEC-6 cells or human FHS-74 cells	Hypoxia/reoxygenation	Human milk	↑(Living cell count, proliferation); ↓apoptosis	[152]
Rat IEC-6 cells	H_2_O_2_	Bovine milk	↑(Cell viability, superperoxide dismutase, glutathione peroxidase); ↓(ROS, LDH, malondialdehyde, NRF2, HO-1)	[153]
Neonate mice intestinal organoids	LPS	Human colostrum, transitional or matured milk	↓(Structural damage, *TNF-α, TLR4, LGR5,* Ki67)	[154]
Neonate mice intestinal organoids	Hypoxia and LPS	Human raw or pasteurized milk	↓(Structural damage, *IL-6*, MPO); ↑(MUC2, goblet cell abundance)	[155]
**In vivo**				
Mice	Non-stimulated	Porcine milk	↑(Small intestinal V/C ratio, *CDX2, PCNA, IGF-1R*); ↓*p53*	[138]
Mice	Non-stimulated	Bovine milk	↑(*MUC2, RegIIIγ, MyD88, GATA4,* IgA, secretory IgA, enterocyte abundance, V/C, cecum surface area)	[156]
Mice	LPS	Porcine milk	↓(IL-1β, IL-6, TNF-α); ↑(jejunum morphology, villi structure, V/C ratio)	[148]
Mice	DON-colitis	Porcine milk	↓(*p53*, *p21,* caspase-3/-9, villi damage); ↑(jejunum villus height, crypt depth, V/C ratio, intestinal length, *β-catenin, CCND1*, phospho-Akt, *ZO-1, OCLN, CLDN1*, miRNAs targeting p53 pathway)	[150]
Mice	DSS-colitis	Human milk	↑(TGF-β1, miRNAs targeting DNMT1/DNMT3); ↓(colon shortening, inflammatory cell infiltration, tissue damage, lesions, *DNMT1/DNMT3*, *IL-6*, *TNF-α*)	[157]
Transgenic mice	Tamoxifen-ulcerative colitis	Bovine milk	↑(Colon length and weight); ↓mucosal injury	[158]
Neonate rats	Formula feeding and hypoxia-induced NEC	Human preterm milk	↑(Villus integrity, enterocyte proliferation); ↑(peptides promoting epithelial proliferation, migration, regeneration and immunomodulation)	[38]
Neonate mice	Formula feeding, LPS and hypoxia-induced NEC	Bovine milk	↓(Intestinal damage, MPO); ↑(MUC2^+^/GRP94^+^ goblet cell abundance)	[147]
Neonate mice	Formula feeding, LPS and hypoxia-induced NEC	Human milk	↓(Intestinal damage, severity and incidence of disease)	[152]
Neonate mice	Formula feeding, LPS and hypoxia-induced NEC	Human raw or pasteurized milk	↑(Goblet/MUC2^+^ cell abundance);↓(MPO, *IL-6*, mucosal injury)	[155]

The arrow indicates an increase (↑) or decrease (↓) in the level or activity of the different parameters analysed, “=” symbol designates unchanged parameters. *Akt* Protein kinase B, *Bax* B-cell lymphoma 2-associated X protein, *CCND1* Cyclin D1, *CDX2* Homeobox transcription factor-2, *CLDN* Claudin, *DNMT* DNA methyltransferase, *DON* Deoxynivalenol, *DSS* Dextran sulphate sodium, *FAS* Cell surface death receptor, *GATA4* GATA binding protein 4, *GRP94* Glucose-regulated protein-94, *HIF-1α* Hypoxia-inducible factor-1α, *HO-1* Heme oxygenase-1, *H*_*2*_*O*_*2*_ Hydrogen peroxide, *Ig* Immunoglobulin, *IGF-1R* Insulin-like growth factor 1 receptor, *IL* Interleukin, *Ki67* Cell proliferation marker, *LDH* Lactate dehydrogenase, *LGR5* Leucine-rich repeat-containing G-protein coupled receptor 5, *LPS* Lipopolysaccharides, *miRNA* microRNA, *MPO* Myeloperoxidase, *MUC2* Mucin 2, *MyD88* Myeloid differentiation primary response 88, *NF-κB* Nuclear factor-κB, *NEC* Necrotizing enterocolitis, *NRF* Nuclear factor erythroid 2-related factor, *OCLN* Occludin, *PCNA* Proliferating cell nuclear antigen, *PHD-1* Prolyl hydroxylases-1, *p-IκBα* phospho-Nuclear factor-κB inhibitor α, *p-NF-κB* phospho-Nuclear factor-κB, *p-NF-κB-p65* phospho-Nuclear factor-κB p65 subunit, *PTEN* Phosphatase and tensin homolog, *Ki67* Cell proliferation marker, *RegIIIγ* Regenerating islet-derived protein 3 gamma, *ROS* Reactive oxygen species, *SERPINE* Serine protease inhibitor clade E, *TGF* Transforming growth factor, *TFF3* Trefoil factor family-3, *TLR* Toll-like receptor, T*NF* Tumour necrosis factor, *Tp53* or *p53* Tumour protein 53, *V/C* Villus height/crypt depth, *VEGFA* Vascular endothelial growth factor-A, *ZO* Zonula occludens

Accordingly, supplementation of porcine MDEs improved the viability and proliferation of IPEC-J2 cells by increasing the gene expression of homeobox transcription factor-2 *(CDX2),* insulin-like growth factor-1 receptor *(IGF-1R),* and proliferating cell nuclear antigen *(PCNA).* Besides, the miRNAs targeting the p53 cell death pathway were transferred from MDEs to the IPEC-J2 cells. This subsequently downregulated the genes involved in p53 pathway such as the *p53,* cell-surface death receptor *(FAS)* and serine protease inhibitor clade E (*SERPINE*) [138]. Likewise, rat MDEs stimulated the viability, proliferation, and stem cell activity of rat IEC-18 cells by activating the gene expression of *PCNA* and leucine-rich repeat-containing G-protein coupled receptor-5 *(LGR5)* [145].

Interestingly, the human MDEs selectively induced proliferation and mesenchymal-like morphology in the human normal CCD841 cells over cancer LS123 cells by modulating the expression of collagen type-I protein and *twist1* gene. Furthermore, tumorigenesis was suppressed only in the normal cells by downregulating the protein expression of phosphatase and tensin homolog [146]. In another human cancer cell model as LS174T, application of bovine MDEs enhanced mucin production by stimulating the expression of genes specific for goblet cell activity such as the *MUC2,* trefoil factor family-3 *(TFF3),* and glucose-regulated protein-94 *(GRP94)* [147]. Following, the MDEs were shown to secure the intestinal barrier from different pathogenic and non-pathogenic stress factors. Accordingly, the human pre-term MDEs highly stimulated the proliferation, migration, and healing of mechanically injured human FHC cells compared to the term-MDEs [38]. In another study, pre-treatment of bovine MDEs protected the IEC-6 cells from the oxidative damage of H_2_O_2_ by enhancing cell proliferation and level of antioxidant enzymes (superperoxide dismutase, glutathione peroxidase). Additionally, the bovine MDEs inhibited the mediators of oxidative stress (ROS, malondialdehyde), LDH release, and the gene expression of *NRF2* and *HO-1* by altering the miRNA profiles of IEC-6 cells [153].

Further, co-administration of porcine MDEs or MDE-miRNAs with LPS decreased the impact of LPS-induced inflammation and apoptosis of IPEC-J2 cells. Particularly, damage on cell viability, genes or proteins activated in TLR4/NF-κB pathway (*TLR4, MyD88*, p-IκBα, p-p65-NF-κB, p-NF-κB, NF-κB), p53 apoptotic signaling *(Tp53, FAS, *Caspase-3) and secretion of pro-inflammatory cytokines (IL-1β/-6, TNF-α) were significantly attenuated [148, 149]. Similarly, in another study by the same group, co-administration of porcine MDEs with DON lowered the impact of DON-induced toxic stress in the IPEC-J2 cells. Specifically, DON-induced damage on cell viability, genes or proteins corresponding to proliferation (β-catenin, cyclin D1 *[CCND1]*, protein kinase B *[Akt])* and tight junction proteins *(ZO-1, OCLN, CLDN1)* were significantly recovered by the treatment of porcine MDEs. Additionally, DON-activated genes or proteins that involved in apoptosis (*Tp53, p21, FAS, SERPINE1*, caspase-3/-9) were suppressed by the porcine MDEs. Besides, the miRNAs targeting the p53 pathway were highly expressed in the IPEC-J2 cells post-incubation with porcine MDEs [150]. In the intestinal organoids of neonatal mice, co-administration of human MDEs (from colostrum, transitional or mature milk) with LPS, markedly reduced the LPS-induced pro-inflammatory gene expression *(TLR4, TNF-α)* and morphological damage. Besides, the human MDEs stimulated the expression of markers specific for epithelial proliferation (Ki67^+^ cells) and regeneration (*LGR5* gene) in the organoids. Amongst the three MDE-treatment groups, colostrum-MDEs exhibited enhanced cytoprotective effects [154].

Several recent studies assessed the capability of MDEs in controlling the hypoxic stress in intestinal barrier. Accordingly, pre-treatment of cow MDEs, yak MDEs or yak MDE-miRNAs enhanced the survival and proliferation (Ki67^+^ cells) of IEC-6 cells under hypoxic stress. Especially, the yak MDEs or MDE-miRNAs enhanced hypoxia resistance by decreasing the protein expression of hypoxia-inducible factor-1α (HIF-1α), vascular endothelial growth factor-A (VEGFA), and different apoptotic markers (p53, B-cell lymphoma 2-associated X protein [Bax], Caspase-3/-9), while upregulated prolylhydroxylases-1 (PHD-1) [39, 151]. Additionally, the miRNA profiling of MDEs has disclosed the presence of miRNAs relevant for hypoxia protection and intestinal barrier development. The microscopic observations also revealed that yak MDEs were highly taken up by the cells during normoxic conditions over the cow MDEs [39, 151]. Likewise, in another study, pre-treatment of human MDEs protected the human FHS-74 and rat IEC-6 cells from alternating hypoxia/reoxygenation injury by inhibiting apoptosis and enhancing the cell proliferation [152]. Similarly, both raw and pasteurized human MDEs reduced the morphologic damage, MPO activity, and the gene expression of pro-inflammatory *IL-6*, while enhanced the goblet cell abundance and MUC2 expression in the hypoxia and LPS-injured neonatal mice intestinal organoids [155].

##### In vivo studies

Previously, administration of porcine MDEs in mice significantly improved the small intestinal morphology (villus height, crypt depth, V/C ratio) and expression of genes and proteins specific for mucosal proliferation (CDX2, IGF-1R, PCNA), while supressed the *p53* [138] (Table 4). Similarly, in another study, bovine MDEs improved the enterocyte abundance and intestinal architecture (villus height, crypt depth, cecum surface area) in mice [156]. In addition, the expression of genes or proteins relevant for mucosal integrity and innate immunity such as the *MUC2, MyD88,* regenerating islet-derived protein 3 gamma *(RegIIIγ),* GATA binding protein-4 *(GATA4),* immunoglobulin A (IgA), and secretory IgA were significantly increased [156]. Further, as observed in cell studies, co-administration of porcine MDEs with LPS ameliorated the severity of LPS-induced inflammation in mice. Particularly, the porcine MDEs suppressing the protein expression of pro-inflammatory cytokines (IL-1β/-6, TNF-α) and improved the small intestinal morphology (villus height, crypt depth, V/C ratio) [148]. Similarly, co-administration of porcine MDEs with DON in mice, reduced the DON-induced damage on intestinal morphology (villus height, crypt depth, V/C ratio), expression of genes and proteins corresponding to jejunal proliferation (β-catenin, CCND1, phospho-Akt), and tight junction proteins *(ZO-1, OCLN, CLDN1)*. The DON-activated genes and proteins that involved in apoptosis (p53, p21, FAS, SERPINE1, caspase-3/-9) were also significantly inhibited [150]. Further, the bovine and human MDEs attenuated the mucosal lesions, lymphocyte infiltration, colon shortening, and gene expression of pro-inflammatory cytokines *(IL-6, TNF-α)* in the DSS-colitis mice. Additionally, the protein level of TGF-β1 and specific miRNAs were upregulated in the mice colon, which subsequently downregulated its target genes as the DNA methyltransferase-1/-3 *(DNMT1/DNMT3)* [157]. Similarly, the bovine MDEs reduced the mucosal inflammation and colon weight/length in the tamoxifen-induced ulcerative colitis in transgenic mice [158].

Moreover, MDEs are able to attenuate the severity of NEC induced by a combination of hypoxia, formula feeding, or LPS challenge in neonatal murine. Accordingly, in NEC-rat pups, supplementation of human pre-term MDEs accumulated in the small intestine, which subsequently increased the ileal villus integrity and enterocyte proliferation. For the first time, peptidomic profiling of MDEs revealed the presence of numerous peptides involved in epithelial proliferation, migration, regeneration, and immunomodulation [38]. Similarly, bovine MDEs improved the distal ileal morphology, goblet cell abundance (MUC2^+^/GRP94^+^ cells) and decreased the MPO expression in the NEC-neonatal mice [147]. Likewise, the administration of human MDEs in the NEC-neonatal mice suppressed the intestinal damage, severity, and incidence of disease [152]. In another study, administration of raw or pasteurized human MDEs in the NEC-neonatal mice, significantly enhanced the goblet cell abundance (MUC2^+^ cells), while attenuated the distal ileal injury, MPO level and, the gene expression of pro-inflammatory *IL-6* [155]. A schematic representation summarizing the anti-inflammatory and antioxidative mechanisms of MDEs through NF-κB inhibition in IEL is reported in Fig. 5.

## Conclusion, limitations, and future perspectives

Based on European Food Safety Authority, the main target of an immunomodulatory feed additive is to stop the local inflammation and prevent further damage to the immune system. According to existing data, it can be concluded that the dietary omega-3 polyunsaturated fatty acids, citrus pectin, and milk-derived exosomes are able to terminate inflammation at the level of the intestinal barrier. The molecular mechanisms of these nutrients in the intestinal barrier are mainly associated with improving the expression of tight junction proteins, epithelial proliferation, enrichment of mucus layer, immunomodulation and prevention of inflammatory cell infiltration. Further, the nutrients support the maintenance of intestinal equilibrium even under stress conditions by enhancing epithelial proliferation and regeneration. Moreover, these nutrients have demonstrated considerable bioaccessibility and bioavailability across the intestinal epithelium, which is a major challenging factor in feed formulations. Although omega-3 polyunsaturated fatty acids are a well-known anti-inflammatory nutrient over decades, till-date its application in controlling IBD is well established in humans, unlike animals. The lack of specific molecular studies at the intestinal level of livestock and poultry is a major setback for achieving desired health outcomes in farm animals. On the other hand, existing data on citrus pectin and milk-derived exosomes are insufficient for harnessing their application as an immunomodulatory feed additive. However, in the future, the novel property of citrus pectin to form gel matrix with mucin and selectively support the intestinal adhesion of probiotics can be utilized to reconstitute the mucosal epithelium that is damaged during infections, antibiotic therapy, or IBD. Additionally, the barrier penetrating property of low molecular weight citrus pectin can be used to target the pro-inflammatory mediators such as Galectin-3 at the local and systemic level. Intriguingly, both omega-3 polyunsaturated fatty acids and miRNA/peptide cargoes of milk-derived exosomes prevent the incidence of necrotizing enterocolitis by suppressing hypoxic stress. They can be excellent nutritional supplements especially in newborn mammals to preventing undesired inflammatory stress, hypoxia, and mortality during the pre- and post-weaning periods. In summary, all these nutrients pose a promising opportunity for controlling chronic inflammatory diseases and promote gut health in farm animals. However, it is noteworthy to mention that the molecular mechanism of these natural bioactive compounds can be diverse and poor understanding can limit its practical application. Therefore, further studies, especially using high-throughput omics technologies are necessary in order to enumerate their precise molecular mechanisms for efficient utilization in animal diets.

## Data Availability

The data used during the current study are publicly available.

## Abbreviations

ALA: Alpha-linolenic acid
ARA: Arachidonic acid
CD: Crohn’s disease
CLDN: Claudin(s)
CPn: Citrus pectin
COX: Cyclooxygenase
DAMPs: Damage-associated molecular patterns
DE: Degrees of esterification
DHA: Docosahexaenoic acid
DM: Degrees of methyl esters
DON: Deoxynivalenol
DSS: Dextran sulphate sodium
EPA: Eicosapentaenoic acid
FAS: Cell-surface death receptor
FD4: Fluorescein isothiocyanate-labelled dextran 4 kDa
Gal-3: Galectin-3
GRP94: Glucose-regulated protein-94
HO-1: Heme oxygenase-1
HRP: Horseradish peroxidase
IBD: Inflammatory bowel diseases
ICAM-1: Intercellular adhesion molecule-1
IEC: Intestinal epithelial cell(s)
IEL: Intestinal epithelial layer
IGF-1R: Insulin-like growth factor-1 receptor
IκB: Inhibitor of nuclear factor kappa B
IL: Interleukin
INFγ: Interferon gamma
iNOS: Inducible nitric oxide synthase
LDH: Lactate dehydrogenase
LGR5: G-protein coupled receptor-5
LT: Leukotriene(s)
LOX: Lipoxygenase
LX: Lipoxin(s)
LPS: Lipopolysaccharides
MaR: Maresin(s)
MCP-1: Monocyte chemoattractant protein-1
MDE: Milk-derived exosome(s)
miRNA: microRNA(s)
MPO: Myeloperoxidase
MUC: Mucin
MyD88: Myeloid differentiation primary response 88
NEC: Necrotising enterocolitis
NF-κB: Nuclear factor-κB
NOD: Nucleotide-binding oligomerization domain-containing protein
NRF2: Nuclear factor erythroid 2-related factor 2
NSAIDs: Anti-inflammatory drugs
ω-3 PUFA: Omega-3 polyunsaturated fatty acid(s)
OCLN: Occludens
PAMPs: Pathogen-associated molecular patterns
PCNA: Proliferating cell nuclear antigen
PG: Prostaglandin(s)
PPARγ: proliferator-activated receptor γ
PRRs: Pattern-recognizing receptors
RIPK: Receptor interacting protein kinase
ROS: Reactive oxygen species
Rv: Resolvin(s)
SCFAs: Short-chain fatty acids
SERPINE: Serine protease inhibitor clade E
TEER: Transepithelial electrical resistance
TGF: Transforming growth factor
TLR: Toll-like receptor(s)
TNBS: 2,4,6-trinitrobenzene sulfonic acid
TNF: Tumour necrosis factor
TXB: Thromboxane(s)
V/C: Villus height/crypt depth
